# Bacterial Communities of *Lamiacea* L. Medicinal Plants: Structural Features and Rhizosphere Effect

**DOI:** 10.3390/microorganisms11010197

**Published:** 2023-01-12

**Authors:** Ekaterina K. Zharkova, Anna A. Vankova, Olga V. Selitskaya, Elena L. Malankina, Natalya V. Drenova, Alena D. Zhelezova, Vitaliy K. Khlyustov, Sergey L. Belopukhov, Aleksey V. Zhevnerov, Ludmila A. Sviridova, Tatiana N. Fomina, Andrey V. Kozlov

**Affiliations:** 1 Russian State Agrarian University-Moscow Timiryazev Agricultural Academy, Moscow 127434, Russia; 2 All-Russian Plant Quarantine Center, Pogranichnaya St. 32, Bykovo, Ramensky District, Moscow 140150, Russia; 3 V.V. Dokuchaev Soil Science Institute, Pyzhyovskiy Lane 7, Building 2, Moscow 119017, Russia

**Keywords:** bacterial communities, rhizosphere, structural features, bacterial diversity, ecological functions, medicinal plants, *Lamiacea* L.

## Abstract

Bacterial communities associated with medicinal plants are an essential part of ecosystems. The rhizosphere effect is rather important in the cultivation process. The purpose of the study was to analyze the rhizosphere effect of oregano (*Origanum vulgare* L.), peppermint (*Mentha piperita* L.), thyme (*Thymus vulgaris* L.), creeping thyme (*Thymus serpillum* L.) and sage (*Salvia officinalis* L.). To estimate the quantity of 16S bacteria ribosomal genes, qPCR assays were used. To compare bacterial communities’ structure of medicinal plants rhizosphere with bulk soil high-throughput sequencing of the 16S rRNA targeting variable regions V3–V4 of bacteria was carried out. The highest bacterial abundance was associated with *T. vulgaris* L., *M. piperita* L. and *S. officinalis* L., and the lowest was associated with the *O. vulgare* L. rhizosphere. Phylum *Actinobacteriota* was predominant in all rhizosphere samples. The maximum bacterial α-diversity was found in *S. officinalis* L. rhizosphere. According to bacterial β-diversity calculated by the Bray–Curtis metric, *T. vulgaris* L. root zone significantly differed from bulk soil. The rhizosphere effect was positive to the *Myxococcota*, *Bacteroidota*, *Verrucomicrobiota*, *Proteobacteria* and *Gemmatimonadota*.

## 1. Introduction

Microorganisms play a significant role in ecosystems [1]. Being involved in various processes, including the circulation of substances, they are ubiquitous [2,3]. Pathogenic microorganisms cause diseases of humans, animals and plants [4]. Using a variety of metabolic processes and having the ability to grow and reproduce even under extreme environmental conditions allows microorganisms to settle on a wide range of natural and synthetic materials in soil, water and air [5]. Microorganisms participate in the biogeochemical cycles of such important elements as C, N, S, P, Fe, Mn, etc. [6]; maintain the structure of the soil; and interact directly or indirectly with plants. Interactions between plants and soil microorganisms can be both mutually beneficial and lead to the death of one or another participant in the symbiosis [7,8]. Microbial communities associated with plants are an important part of ecosystems [9]. Symbiotic microorganisms are able to protect the host plant from pathogenic microorganisms and insect pests, thereby improving the quality of crop production [10,11]. Among positive effects, the following facts of mutual influence can be noted: prokaryotes and micromycetes provide plants with nitrogen, promote the absorption of phosphorus and synthesize auxins and phytohormones, stimulating root growth [9,12]. Increased microorganisms’ activity is observed in the rhizosphere, which contains a significant amount of root exometabolites. Biologically active substances of plant origin can enter the upper layers of the soil not only with root exudates but also with fallen leaves and remnants of stems washed off during precipitation, as well as coming with gaseous products [13]. During the growing season, according to various data, the plant releases up to 30–50% of the carbon obtained as a result of photosynthesis into the soil [9]. The biological meaning of such a large-scale energy release cannot be understood outside of microbial–plant interaction. However, plant metabolism products can either stimulate the growth and development of microorganisms and plants or produce an allelopathic effect, as well as contributing to soil fatigue during long-term cultivation in the arable layer of soils [14]. Due to the synthesis of substances with high biological activity, essential oil plants form phytogenic fields around their place of growth [15]. Rhizosphere microorganisms in such areas varied from microbial communities of the bulk soil. The long-term essential oil plant cultivation leads to phytopathogenic micromycetes growth, with the toxins produced by soil microorganisms increasing greatly. All these factors are considered to form an allelopathic soil regime, resulting in soil fatigue [16].

Because of the fact that essential oil plants form powerful phytogenic fields and stimulate soil due metabolites entering the soil, the rhizosphere effect is usually common to medicinal crop plantations [17]. The rhizosphere effect results from biological, chemical and physical changes in soils that occur because of root cap and border cell loss; the death and lysis of root cells; the flow of C to mycorrhizas and other root-associated symbionts living in the soil; gaseous losses; the leakage of solutes from living cells known as root exudates; insoluble polymer secretion, such as mucilage from living cells; the loss of C due to the death and lysis of root epidermal and cortical cells; flushing from shoots; and leave and stem deposits [18,19,20]. Therefore, the study and regulation of microbial communities associated with the rhizosphere of medicinal plants are important practical tasks [21,22]. The commercially significant medicinal plants of the *Lamiaceae* L. family, such as oregano, peppermint, thyme, creeping thyme and sage, are known to accumulate essential oil in the aboveground plant parts, and the latter is recognized as a promising antimicrobial agent [23].

Being the most extensively studied in traditional medicine, the *Thymus* genus of the *Lamiaceae* family is famous for having both antimicrobial and anti-inflammatory activity, as well as spasmolytic, immunomodulatory and antioxidant capabilities [24]. There are about 350 *Thymus* species in Europe, Asia and North Africa; among them *Thymus vulgaris* L. is the typical genus member used for thousands of years in agriculture [25]. Evergreen leaves of this aromatic plant accumulate essential oil, which consists of thymol, geraniol, linalool, borneol and carvacrol, the main active components varying across chemotypes [23].

Comprising one more important representative of the *Lamiaceae* family, *Origanum* genus is widely distributed in Europe, North Africa and Asia under low land and mountainous environments [26]. Oregano (*Origanum vulgare* L.) is a woody-based perennial plant that was noted by Hippocrates as an antiseptic agent [27]. Essential oil glands located on oregano hairy leaves contain terpinen-4-ol, *cis*-sabinene, *o*-cymene, g-terpinen, *trans*-sabinene and other compounds with spasmodic, antimicrobial, digestive, expectorant and aromatic properties [25]. The *Origanum* is used to cure diabetes, insomnia, catarrh and asthma; the application features depend on the component compositions and peculiarities of the diagnosis [28].

The *Salvia* genus from the *Lamiaceae* family incorporates the oldest medicinal plant used by humans in the Mediterranean area [25]. It is considered to be the panacea for its antibacterial, antiviral, antioxidative, antimalarial, anti-inflammatory, antidiabetic, cardiovascular and antitumor properties [29]. Sage (*Salvia officinalis* L.) essential oil contains more than 100 active substances categorized into monoterpene hydrocarbons, oxygenated monoterpenes, sesquiterpene hydrocarbons, diterpens, oxygenated sesquiterpenes and nonisoprenoid compounds [30]. Rosmarinic acid is the major leaf component; 1,8-cineole, camphor and a variety of thujenes are the most abundant in sage essential oil [31,32].

The *Lamiaceae* family medicinal plants comprise of 12 to 18 *Mentha* species widely distributed across Europe, Asia, Africa, Australia and North America [33]. The most common for commercial cultivation is *Mentha piperita* (peppermint), having been known as the hybrid of *M. spicata* and *M. aquatica* since the time of the ancient Egyptians [25]. The list of benefits of peppermint associates with its phytochemical features of active substances varying on maturity stage, variety, geographical zone and processing methods [34]. The volatile components are menthol, menthone, isomenthone, eucalyptol and limonene; the fatty acids are represented by palmitic, linoleic and linolenic acids [35]. The maximum quantity of active substances is usually obtained in the full flowering stage, varying on the growing conditions. Traditional uses of peppermint are the therapy of biliary maladies, enteritis, gastritis, intestinal colic and spasms of the gastrointestinal tract [34].

The highest metabolic activity of essential oil of *Lamiaceae* plants is obtained during flowering periods [36]. In case the plantation is infected with phytopathogenic microorganisms, the composition of essential oil deteriorates and its amount decreases [25,26]. To estimate non-phytopathogenic bacterial communities influenced by biologically active substances in the rhizosphere, the healthy flowering plants are recommended to be used [37]. Not all bacterial species are cultured in the traditional way so the high-throughput sequencing and PCR methods are applied to amplify 16S ribosomal bacteria genes [38,39].

Sequences are typically clustered by similarity to generate operational taxonomic units (OTUs). Representative OTU sequences are compared with reference databases for taxonomy revealing [40]. To analyze bacterial metagenome, the following biodiversity indices are used. The Shannon and Chao1 indexes characterize α-diversity. Chao1 index is quite informative, which allows estimations of the actual number of taxa in the sample and takes into account taxa with one and two sequences, obtaining a more reliable picture of the taxa number in the community [41]. The Bray–Curtis coefficient makes it possible to estimate β-diversity. If two bacterial communities are completely identical, this indicator is equal to 0, and the complete absence of similarity is equal to 1 [42].

Certain types of bacteria noticeably promote growth and development of plants known as plant growth promotion rhizobacteria (PGPRs) [43]. The study of such plant-friendly bacteria, including NGS methods, form the basis of increasing the productivity of medicinal plants, improving their resistance to pests and diseases and reducing the pesticide load, as well as, furthermore, protecting plant growers’ and consumers’ health, alongside that of the planet as a whole.

## 2. Materials and Methods

### 2.1. Sampling

**Figure 1:**
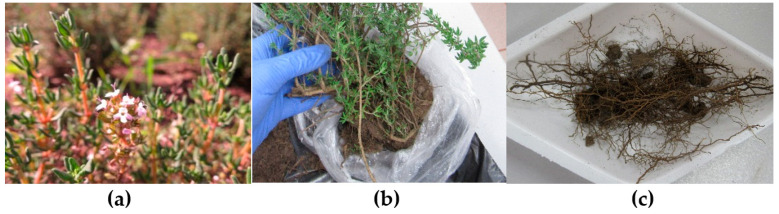
Stages of sampling: (**a**) healthy flowering plants selecting; (**b**) the plant with soil monolith collected; (**c**) rhizosphere with roots.

The objects of the study were the rhizosphere samples of the following medicinal plants oregano (*Origanum vulgare* L.), peppermint (*Mentha piperita* L.), thyme (*Thymus vulgaris* L.), creeping thyme (*Thymus serpillum* L.) and sage (*Salvia officinalis* L.), cultivated at the plots of the collection site of the Educational, Research and Production Center of Horticulture and Vegetable Growing, named after V. I. Edelstein in RSAU-MTAA (55°49′36.5″ N 37°33′05.0″ E). The rhizosphere was used. Mature flowering plants with soil monoliths were placed into sterile plastic bags using sterile gloves, then cool delivered to the laboratory at the same day (Figure 1).

All species were collected in 5 replicates at a distance of 1.5–2 m from each other. Plants were grown in medium sod-podzolic loamy well-cultivated soil (pH_KCl_—6.2, total organic carbon—2.92%, total nitrogen—141 mg/100 g, P_2_O_5_—285 mg/kg, K_2_O—250 mg/kg) without rotation for 7 years. Immature roots (2 mm thick) were used to obtain rhizosphere by careful shaking [13].

Bulk soil at a depth of 10 cm was used as control sample. Chemical analysis was carried out according to standard methods [44]. Carbon level was measured as TOC (total organic carbon) with CNSoft at VELP CN 802 elemental analyzer (Velp Scientifica, Usmate Velate, Italy). Moisture was defined with Sartorius MA (Sartorius MA, Goettingen, Germany), pH was calculated with pH conductometer (Mettler Toledo, Greifensee, Switzerland).

### 2.2. Extraction of DNA

Each soil sample (0.5 g of fresh soil, delivered on ice from plant plots at 30 min) was homogenized in Tissue Lyser II (QIAGEN, Hilden, Germany) at 30 Hz for 3 min. MetaGen reagent kit (Syntol, RF) was used to extract total DNA, as recommended by the manufacturer. To estimate DNA quality, the electrophoresis in agarose gel (1% *w/v* in TBE) was used with visual DNA detection with Gel Doc XR+ System (Bio-Rad Laboratories, Hercules, CA, USA). Quantity of DNA was measured by Nano-500 (Allsheng, PRC, Hangzhou, China).

### 2.3. The qRT-PCR Analysis

The qPCR assays were used to estimate quantity of 16S ribosomal genes of bacteria. Primer sets for quantifying were Eub338 (ACTCCTACGGGAGGCAGCAG) and Eub518 (ATTACCGCGGCTGCTGG) [45]. For the construction of standard curves for PCR products of total bacteria, total soil DNA was used. The qPCR mix contained 2.5 µL of dNTP, 2.5 µL 10 × PCR buffer, 2.5 µL MgCl_2_, SynTag DNA-polymerase (Syntol, RF), 0.5 µM of each primer and 1 µL of extracted soil DNA template in a total volume of 25 µL. PCR conditions were 3 min at 95 °C, followed by 49 cycles of 95 °C for 10 s, 50 °C for 10 s and 72 °C for 20 s carried out in C1000 Thermal Cycler with the CFX96 Real-Time System (Bio-Rad Laboratories, Hercules, CA, USA) [45]. Melting curve analysis was applied (65–95 °C, increment—0.5 °C) to ensure qPCR quality. Standard curves ranged from 10^2^ to 10^7^ gene copy number/µL in triplicate. Efficiencies of qPCR were above 88.8, the coefficient of determination was R^2^ > 0.98 for all standard curves.

### 2.4. Sequencing Analysis

For each studied soil high-throughput sequencing of the 16S rRNA, gene libraries were realized. The purified DNA isolates were amplified with universal multiplex primers F (TCGTCGGCAGCGTCAGATGTGTATAAGAGACAGCCTACGGGNGGCWGCAG) and R (GTCTCGTGGGCTCGGAGATGTGTATAAGAGACAGGACTACHVGGTATCTAATCC), targeting 16S V3–V4 region of bacteria 16S genes, according to standard methods [46]. The 16S rRNA gene libraries sequencing were carried out on DNBSEQ G-50, according to manuals of manufacturer. The raw data were processed using QIIME. Sequences were filtered in Trimmomatic program. Nucleotide similarity of 97% was used to form operational taxonomic units (OTU). VSEARCH algorithm was filtered chimeras, and SILVA database was served as reference system. Singletons were excluded. Data obtained were visualized with MS Excel 2016.

### 2.5. Biodiversity Analysis

Statistical analysis of gene abundance data was performed in STADIA 6.0 (RF). Several indices were used for the estimation of total diversity of the studied bacterial communities (α-diversity). The Shannon and Chao1 indexes were calculated to characterize the real number of bacterial community members. Structural differences between bacterial communities (β-diversity) were analyzed using binary metric of similarity—Bray–Curtis metric [32,33].

## 3. Results

### 3.1. Rhizosphere and Control Samples Properties

Certain chemical properties of samples studied were different. *O. vulgare* L. had the driest rhizosphere. *M. piperita* L. had the highest moisture percent. *T. vulgaris* L. and *O. vulgare* L. rhizosphere were rich in total organic carbon and, at the same time, *T. serpillum* L. had the poorest one. The pH values ranged from 7.12 to 7.37 meanings. DNA quantity varied from 33.1 μg/g in *O. vulgare* L. rhizosphere samples to 57.5 μg/g in *M. piperita* L. rhizosphere soil (Table 1).

### 3.2. Bacterial Gene Abundance

**Figure 2:**
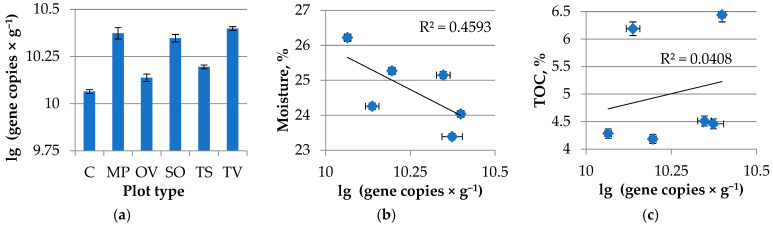
(**a**) Bacterial gene abundance in *Lamiacea* L. medicinal plants rhizosphere. MP is peppermint (*M. piperita* L.), OV—oregano (*O. vulgare* L.), TV—thyme (*T. vulgaris* L.), TS—creeping thyme (*T. serpillum* L.), SO—sage (*S. officinalis* L.), C—control; (**b**) Bacterial gene abundance and soil moisture correlation; (**c**) Bacterial gene abundance and TOC correlation. R^2^—accuracy of approximation.

The amount of bacterial gene copies varied from sample to sample. There were two groups clustered from bacterial abundance in rhizosphere: the highest one was obtained in the *T. vulgaris* L., *M. piperita* L. and *S. officinalis* L.; *O. vulgare* L. and *T. serpillum* L. rhizosphere formed another cluster. Control samples differed significantly between rhizosphere samples (Figure 2).

There was no significant linear correlation obtained in terms of bacterial gene abundance and moisture or total organic carbon with the rhizosphere samples tested. However, there was a tendency for the lower moisture and higher carbon content in the soil samples to be more abundant bacterial genes in the rhizosphere and control soil.

### 3.3. Taxonomic Structure of Bacterial Communities in Rhizosphere and Control

The microorganisms of the rhizosphere of *Lamiaceae* L. medicinal plants differ from each other and from the bacterial communities of the bulk soil. High-throughput sequencing of the 16S rRNA targeting variable regions V3–V4 of bacteria revealed the structure of bacterial communities in the rhizosphere and the bulk soil. In total, 2086 sequences of the 16S rRNA gene were obtained (340–360 sequences per sample), with a mean length of 465 bp.

**Figure 3:**
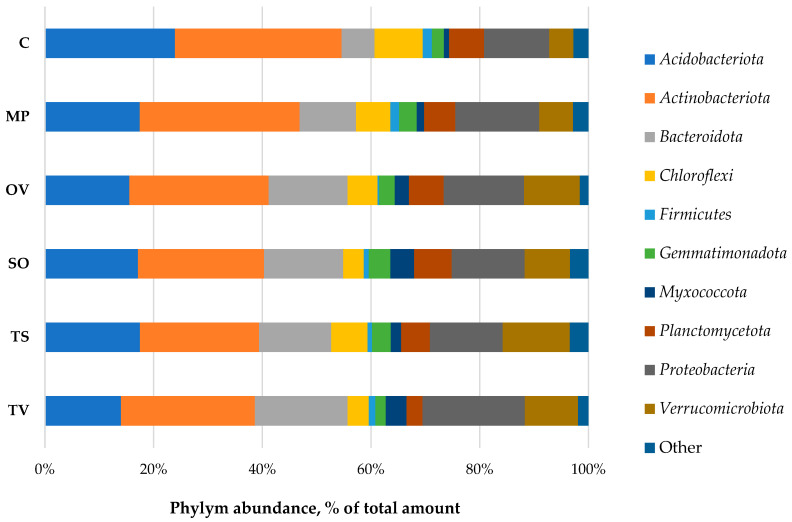
Taxonomic structure of bacterial phyla in *Lamiaceae* L. rhizosphere. MP is peppermint (*M. piperita* L.), OV—oregano (*O. vulgare* L.), TV—thyme (*T. vulgaris* L.), TS—creeping thyme (*T. serpillum* L.), SO—sage (*S. officinalis* L.), C—control. ‘Other’ means phyla with abundance lower than 1% of total amount.

Bacteria domain was presented by 24 phyla; the dominant domains (more 1% of all bacteria taxa) were *Acidobacteriota*, *Actinobacteriota*, *Bacteroidota*, *Chloroflexi*, *Firmicutes*, *Gemmatimonadota*, *Myxococcota*, *Planctomycetota*, *Proteobacteria* and *Verrucomicrobiota* (Figure 3).

**Figure 4:**
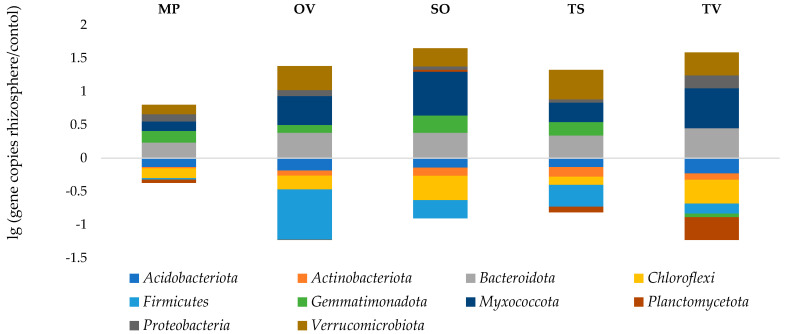
Rhizosphere effect. MP is peppermint (*M. piperita* L.), OV—oregano (*O. vulgare* L.), TV—thyme (*T. vulgaris* L.), TS—creeping thyme (*T. serpillum* L.), SO—sage (*S. officinalis* L.), C–control.

Phylym *Actinobacteriota* were predominant in all samples (23.2–30.7%). There were no significant correlations found between phylum abundance, TOC and moisture. The ratio of the OTU number in the rhizosphere to that in the bulk soil, called the rhizosphere effect, was negative to the *Acidobacteria*, *Chloroflexi* and *Firmicutes*. The positive one, especially formed for *Bacteroidota*, *Myxococcota* and *Verrucomicrobiota* phyla, possibly indicates a symbiotic relationship of the medicinal plants and bacteria phyla detected. *Gemmatimonadota* was abundant in the rhizosphere of all plants tested, but in thyme, rhizosphere was depressed slowly. *T. vulgaris* L. also downsized *Planctomycetota* phylum in its rhizosphere (Figure 4).

### 3.4. Biodiversity

The maximum number of operation taxonomic units (OTU) was obtained in *S. officinalis* L. rhizosphere. It was 1.4 times higher than the bulk soil. Contrariwise, thyme species (*T. vulgaris* L. and *T. serpillum* L.) were 1.1 times lower than the total OTU number in rhizosphere in comparison with bulk soil. α-diversity of rhizosphere bacterial communities, calculated as Chao I and Shannon indices, varied from sample to sample. The highest OTU diversity was obtained among the *S. officinalis* L. roots (Chao I index was 1.5 times higher in comparison with control; Shannon index exceeded the control by 1.1 times). *T. vulgaris* L. was the most powerful influencer for soil bacterial communities: α-diversity was limited 0.7 times for Chao I index, and β-diversity (Bray–Curtis metric) value was 0.51 in comparison with control soil (Table 2).

**Figure 5:**
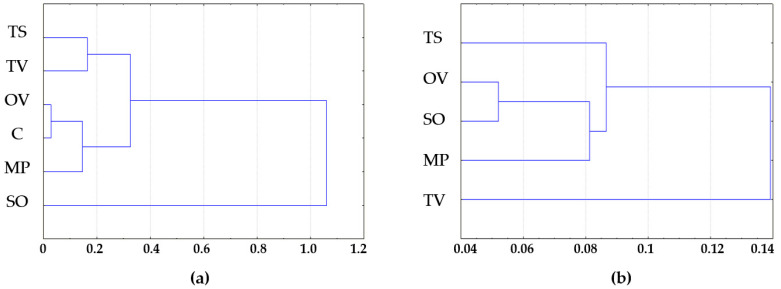
(**a**) Bacterial α-diversity—Shannon and Chao1 indexes; (**b**) Bacterial β-diversity—Bray–Curtis metrics clusters by Euclidean distance. MP is peppermint (*M. piperita* L.), OV—oregano (*O. vulgare* L.), TV—thyme (*T. vulgaris* L.), TS—creeping thyme (*T. serpillum* L.), SO—sage (*S. officinalis* L.), C—control.

The rhizosphere samples tested were grouped into clusters by Euclidean distance in STADIA 6.0. The *S. officinalis* L. rhizosphere samples form a separate cluster from α-diversity indexes, and *T. vulgaris* L. significantly differed from other samples tested by bacterial β-diversity, calculated as the Bray–Curtis metric in comparison with control soil (Figure 5).

According to clustering analysis, *S. officinalis* L. formed the most diverse bacterial communities in rhizosphere when *T. vulgaris* L. had the highest selective activity for bacterial taxa near its roots.

## 4. Discussion

The structure of bacterial communities in the rhizosphere of medicinal plants influence the introduction and cultivation of plants greatly. It has been found that the bacteria in the rhizosphere of the studied *Lamiaceae* plant species were mainly represented by the following phylum: *Acidobacteriota*, *Actinobacteriota*, *Bacteroidota*, *Chloroflexi*, *Firmicutes*, *Gemmatimonadota*, *Myxococcota*, *Planctomycetota*, *Proteobacteria* and *Verrucomicrobiota*. In total, they made up 97.1–98.4% of bacterial communities. The mostly abundant phylum was *Actinobacteriota* (23–31% of the total number of sequences), then followed by *Acidobacteriota* (14–24%), *Proteobacteria* (12–19%), *Bacteroidota* (6–17%) and *Verrucomicrobiota* (5–12%). The representatives of *Chloroflexi*, *Gemmatimonadota*, *Planctomycetota* and *Myxococcota* did not exceed 10% of the total number of sequences. The number of *Firmicutes* was low, up to 1–2%.

Bacterial communities in the rhizosphere, regardless of the plant type, were characterized by actinobacteria dominance. In accordance with generally accepted data, the *Actinobacteriota* phylum includes G+ mycelial and unicellular prokaryotes that are very diverse in morphology and physiology. Actinobacteria are capable of using all known variants of energy and constructive exchange with the exception of phototrophs. For chemoorganotrophs of the respiratory type, the main ecological niche is the aerobic zone of the soil, including the rhizosphere. Their number can reach 10^6^ CFU/g calculated conventionally. These are mainly higher actinobacteria, for example, representatives of *Streptomyces* genus [47]. Actinobacteria secrete a huge amount of exoenzymes, causing rapid increase in biological activity in the root zone. They occupy an important place in the biological cycles, especially in the soil, acting primarily as destructors of organic substances. Many representatives of this group are able to produce phytohormones, antibiotics and other biological compounds, which allows bacteria to enter into various relationships with plants [48].

The phylum *Acidobacteriota* were next in the number of sequences after *Actinobacteriota*. The participants of this phylum are known to be small rod-shaped bacteria with the G-morphotype. Based on DNA sequences, they are extremely widespread [49,50,51]. In the soil, representatives of the genus Acidobacterium are known as chemoorganoheterotrophs with oxidative metabolism, using starch, sugars, organic acids (acetate, lactate, propionate, succinate, fumarate) or aromatic compounds as substrates. They are usually mesophiles, moderate acidophiles and are mostly uncultivated [47].

The phylum *Proteobacteria* is the most extensive group of G- bacteria, diverse in morphology and physiological functions. In the rhizosphere of non-leguminous plants, α- and γ-proteobacteria and representatives of the *Aeromonas*, *Alcaligenes*, *Azospirillum*, *Azotobacter*, *Citrobacter*, *Enterobacter*, *Esherichia*, *Klebsiella*, *Pantoea*, *Pseudomonas* and *Xanthobacter* are quite widespread [47]. Proteobacteria are typically heterotrophs, facultative anaerobes, tolerating of a rather low pH value and generally found under forest plantations, herbaceous non-leguminous plants and vegetable crops cultivated on podzolic soils of temperate climate zone. There are many members of the genera that are capable of stimulating plant growth and development (PGPR), increasing plant resistance to biotic and abiotic stresses due to nitrogen fixation, increasing availability of nutrients and producing phytohormones, antibiotics and other extracellular metabolites [52,53,54,55,56]. The rhizosphere bacterial activity is determined by the amount of organic substances available in the plant root zone, which, in turn, is due to the speed of photosynthesis.

Members of the *Bacteroidota* phylum are G-rods of different morphology and size and chemoorganoheterotrophs with a respiratory or fermentation-type metabolism. They transform carbohydrates, cellulose and peptides. Representatives of the classes *Flavobacteria* and *Sphingobacteria* are widely distributed in soils, silts, phyllosphere and rhizosphere [17]. The bacteria of the *Bacteroides* class live in the digestive tract of insects and mammals; they are obligate anaerobes [22]. In the rumen of ruminants, they cause the decomposition of cellulose to glucose, which is then fermented to form organic acids, alcohols and gaseous products. Bacteroides are found in the phyllosphere of cereal forage grasses and silage and come in the animal’s body with forage [57]. Cellulose-decomposing bacteria are practically absent in the rhizosphere of young plants and appear at later stages of their development. Obviously, the source of nutrition for them is not root exudates but dying cells of root epidermis.

Members of the phylum *Verrucomicrobiota* are G-rods or ovoid cells that are often equipped with prostheca. They are known as chemoorganoheterotrophs using a variety of sugars, but they are incapable of utilizing alcohols, amino acids and organic acids. Verrumicrobia are extremely widespread in soils, rhizosphere, freshwater or marine reservoirs and in human digestive tract [58,59,60,61]. They are one of the most dominant in the composition of prokaryotic communities of soils and are mostly uncultivated.

As a result of the research, the data of the rhizosphere effect for all five studied species of medicinal plants were revealed. *Myxococcota*, *Bacteroidota*, *Verrucomicrobiota* and *Proteobacteria* dominated in all rhizosphere samples. This corresponds to the data obtained in other studies. *Myxococcota* are Gram-negative, mainly aerobic sticks, that habituate in the soil, which are characterized by a complex development cycle, high socialization and the ability to form fruiting bodies. Having a sliding motion due to dense mucous cords, these bacteria are characterized as chemoorganoheterotrophs, some representatives being able to decompose cellulose and chitin, as well as being able to form antibiotic resistance [62,63]. This bacterial taxon is considered more abundant in the rhizosphere than outside of it. In the studied samples of medicinal plants rhizosphere, the greatest rhizosphere effect for bacteria of this phylum was observed in sage, thyme and oregano. The root zone of mint and creeping thyme was less attractive for these bacteria.

*Bacteroidota* exhibited a pronounced rhizosphere effect in all species of medicinal plants under study. The maximum amount of bacteria of this phylum was found in common thyme; slightly less in sage, oregano and creeping thyme; and least of all in peppermint. Flavobacteria and sphingobacteria, belonging to the *Bacteroidota* phylum, are known to be typical soil bacteria and common in the plant rhizosphere. Many members of the Flavobacteria class exhibit growth-stimulating and antibiotic activity [64]. The presence of the genus *Cytophaga* in the *Bacteroidota* phylum, known as active cellulolytics, was found in various types of soils, litter and rhizosphere of many plants [65]. Since the perennial plants were grown without rotation for 7 years, there were a lot of dead cells in rhizosphere as a source of nutrient substrate for *Cytophaga*.

*Verrucomicrobiota* is another phylum of Gram-negative bacteria, which characterized a well-cultivated soil [27,30]. The rhizosphere effect on this bacteria phylum was shown in all samples studied, but their number varied significantly depending on the cultivated plant. The smallest amount of *Verrucomicrobiota* was in the peppermint rhizosphere. Sage, thyme and oregano stimulated the development of representatives of this phylum. By metabarcoding, it was found that verrumicrobia were in the upper horizons of the soil rich in organic matter [66]. It can be assumed that these bacteria are stable or capable of utilizing phenolic compounds synthesized by plants of this type.

In the rhizosphere of all the studied plant species, an increase in Proteobacteria was noted. The greatest rhizosphere effect was obtained in thyme samples. Proteobacteria is known by their catabolic versatility, effective ability to colonize roots and the production of a wide range of enzymes and metabolites that help plants withstand various biotic and abiotic stresses. For example, the genus *Pseudomonas* are widely distributed in various types of soils and the root zone of plants, representatives of this genus have the ability to suppress pathogens and stimulate the growth of crops [67]. *Pseudomonas putida*, in particular, produces ACC deaminase, which promotes the development of a stronger root system and leads to increased plant resistance to stress [68].

*Gemmatimonadota* exhibits a relatively weak rhizosphere effect in all studied medicinal plants. The most decreased number of *Planctomycetota* was observed in the root zone of thyme. However, the proportion of these phylum in microbiomes was not large.

A negative effect of the rhizosphere was obtained for *Acidobacteria*, *Actinobacteria*, *Chloroflexi* and *Firmicutes* in the root zone of all studied medicinal plants. The *Chloroflexi* are typically spread in anaerobic habitats and play an important enzymatic role in the decomposition of complex polymer organic substances to support the growth of bacterial populations [34]. *Firmicutes* are G+ bacteria with endospore-forming ability, varying in energy and constructive exchange. They habituate under various soil conditions and usually form a negative rhizosphere effect in plant root zones [27,35]. *Firmicutes* possess an exceptionally wide collective norm of genotype response, determining high adaptability. Due to members’ diversity, high abundance and wide distribution, *Firmicutes* play one of the main roles in the biosphere. They control the cycles of biogenic elements together with actinobacteria, proteobacteria and cyanobacteria.

The number of identified operating units (OTU) in the samples studied was not too high and ranged from 133 to 202. If we compare this indicator with the values obtained by other researchers, then for the upper horizons of sod-podzolic soils, the number of identified operational units was more than 400 [69]. There was only 143 units in the bulk soil without plants studied. Bacteria diversity in the rhizosphere was not significantly changed for oregano and peppermint, then medicinal sage and thyme of both species had a more noticeable effect on the diversity in comparison with control soil microbial community. In the thyme rhizosphere, OTU was lower than the control soil, and the highest value was found in the sage rhizosphere—202.

The Shannon index is most often used to characterize alpha diversity. This indicator varies in 4.6–8.4 for sod-podzolic soil horizons [70]. According to this indicator, all samples were characterized by a fairly high species diversity. The Shannon index of microbial communities in the rhizosphere of all the studied plants was in the range 7.58 to 8.58. However, all plants except sage affected the diversity of microorganisms in the rhizosphere in the decreasing direction. To estimate the actual number of taxa in the samples studied, the Chao1 index was calculated in a range of 240–485 in comparison with 133–202 OTU.

The beta-diversity was submitted by the Bray–Curtis coefficient. The greatest similarity of bacterial communities in the rhizosphere and soil without plants was revealed for peppermint, followed by medicinal sage, common oregano and creeping thyme. Common thyme had the greatest microbial community formation effect of the rhizosphere.

The microorganisms of the rhizosphere of *Lamiaceae* L. medicinal plants differed from each other and from the bacterial communities of the bulk soil. The most dissimilar to the control samples were S. officinalis L. and T. vulgaris L. rhizosphere. This may be due to the fact that both plants were introduced for the sod-podzolic soil zone. It is known that introduced plants forming exometabolites differ significantly from local plant species. So, bacterial consumers of root exudates and plant wastes vary from the local biota.

## 5. Conclusions

The conducted studies revealed differences between the structure of bacterial communities of the rhizosphere of medicinal plants and a control sample of zonal sod-podzolic soil. If the bacterial phylum were identical in samples, their ratio was different. Positive rhizosphere effect for all five studied species of medicinal plants of the *Lamiaceae* family was obtained for the phylums *Myxococcota*, *Bacteroidota*, *Verrucomicrobiota*, *Proteobacteria* and *Gemmatimonadota*. The rhizosphere microbiomes of five plant species were decreased in *Acidobacteriota*, *Actinobacteriota*, *Chloroflexi* and *Firmicutes*. Medicinal plants formed and controlled their microbiome, creating a favorable ecological niche for bacteria capable of providing the most effective interaction. It should be noted that the positive rhizosphere effect was revealed only for G- bacteria, while G+ decreased their presence in the root zone of plants studied. The reduction in the proportion of G+ bacteria may be due to their greater sensitivity to phenolic compounds synthesized by *Lamiaceae* herbs. At the same time, many representatives of *Actinobacteriota* and *Firmicutes* were important components of the saprotrophic hydrolytic complex, actively participating in the decomposition of complex organic compounds. Therefore, a decrease in their active participation in the microbiome of the rhizosphere can lead to a delay in the transformation processes. The greatest influence on the microbiome formation was exerted by species introduced in the zone of sod-podzolic soils *S. officinalis* L and *T. vulgaris* L., which may be due to their exometabolites that are significantly different from local plant species.

## Figures and Tables

**Table 1:** Rhizosphere and control samples properties.

Plot Type	pH (H_2_O)	Moisture, %	TOC, %	DNA Quantity, μg/g
*Mentha piperita* L. (MP)	7.37	26.22	4.46	57.50
*Origanum vulgare* L. (OV)	7.12	23.39	6.19	33.10
*Salvia officinalis* L. (SO)	7.24	24.04	4.51	54.03
*Thymus serpillum* L. (TS)	7.18	24.26	4.19	34.12
*Thymus vulgaris* L. (TV)	7.14	25.27	6.44	41.98
Control (C)	7.19	25.15	4.29	44.15

**Table 2:** Diversity indices of microbial communities in *Lamiaceae* L. medicinal plants rhizosphere.

Plot Type	OTU Number	Chao I	Shannon	Bray–Curtis (Rhizosphere vs. Control)
*Mentha piperita* L. (MP)	149	300	7.87	0.3
*Origanum vulgare* L. (OV)	144	313	7.96	0.38
*Salvia officinalis* L. (SO)	202	485	8.58	0.35
*Thymus serpillum* L. (TS)	133	266	7.68	0.43
*Thymus vulgaris* L. (TV)	133	240	7.59	0.51
Control (C)	143	321	7.97	-

## Data Availability

Research data was presented in the tables and figures in the main text of the article.
